# Quality of life (QoL) for people with primary sclerosing cholangitis (PSC): a pragmatic strategy for identifying relevant QoL issues for rare disease

**DOI:** 10.1186/s41687-022-00484-5

**Published:** 2022-07-15

**Authors:** Elena Marcus, Patrick Stone, Douglas Thorburn, Martine Walmsley, Bella Vivat

**Affiliations:** 1 Population Health Research Institute, St George’s University of London, Cranmer Terrace, London, SW17 0RE UK; 2 Marie Curie Palliative Care Research Department, Division of Psychiatry, University College London, London, UK; 3 University College London Institute of Liver and Digestive Health, UCL Royal Free Campus, Royal Free Hospital, London, UK; 4 Sheila Sherlock Liver Unit, Royal Free Hospital, London, UK; 5 PSC Support, Didcot, UK

## Abstract

**Background:**

Primary sclerosing cholangitis (PSC) is a rare incurable disease of the bile ducts and liver which can significantly impair quality of life (QoL). No existing QoL tools are entirely suitable for people living with PSC (PwPSC). We aimed to develop a measure of QoL for PwPSC in the UK, beginning by identifying relevant QoL issues. This paper describes our approach to this first stage, and discusses related benefits and limitations.

**Methods:**

Scientific consensus on how to reliably stage PSC is lacking, due to its rarity and heterogeneity. We initially hypothesised four categories for PSC severity. After beginning the study, these were revised to six. For such a rare disease, the study could not recruit sufficient participants in each of these categories, particularly the more severe, in the time available. We therefore modified the design, adapting standard methodology for identifying potentially relevant issues. We started by conducting a thematic analysis of data from a previous survey of PwPSC, and extracting QoL issues from a literature review of QoL questionnaires of relevance to PwPSC. We then conducted group and individual interviews with PwPSC and clinicians, investigating the relevance, importance, phrasing, and breadth of coverage of issues identified. We also explored the validity of our hypothesised categories for disease severity.

**Results:**

We identified 1,052 potentially relevant QoL issues from the survey and literature review and took 396 of these forwards for discussion with 28 PwPSC. We found 168/396 issues were considered relevant by ≥ 60% of these participants. We then discussed this subset of 168 issues with 11 clinicians. PSC and clinician participants identified some problematic phrasing with 19 issues, due to potential upset (n = 12) or problems with understanding (n = 7). We included one new issue from those suggested.

**Conclusion:**

We identified a range of QoL issues relevant to PwPSC, with a good breadth of coverage, although lacking an in-depth understanding of the PSC experience. Our strategy effectively identified relevant QoL issues for people living with this rare condition, for which there is no consensus on stratifying for its severity. This strategy should however be considered specific to such circumstances, not a general recommendation for an alternative approach.

**Supplementary Information:**

The online version contains supplementary material available at 10.1186/s41687-022-00484-5.

## Background

Primary sclerosing cholangitis (PSC) is a rare, progressive, and incurable disease of the bile ducts and liver [1], highly co-morbid with inflammatory bowel disease (IBD), and associated with increased risk of colorectal and hepatobiliary cancers [2, 3]. A UK population-based study estimated annual PSC incidence as 0.68/100,000 person-years and point prevalence of 5.58/100,000 person-years [4]. People with PSC (PwPSC) can experience a variety of symptoms and functional impacts including fatigue, itch, pain, and weight loss [5]. PSC, like many rare diseases, has a highly variable clinical course, characterised by fluctuating liver biochemistry test results and symptoms which pose challenges in staging the condition [6]. With a lack of curative medical treatment, PwPSC can live for many years with debilitating symptoms and uncertainty about disease progression [7], all of which can impact quality-of-life (QoL) [8].

It is important to include patient-reported outcomes (PROs), such as QoL, in clinical research to ensure that the safety and efficacy of new treatments is assessed holistically [9]. Our recent review found very few PSC clinical trials which assessed QoL [10]. In our review we also observed that generic QoL questionnaires may lack sensitivity and fail to measure important impacts of PSC, such as fatigue, and that surrogate endpoints commonly used in trial settings (e.g. serum alkaline phosphatase or bilirubin) may not correlate with QoL. The development of a reliable and valid disease-specific QoL measure would help address these limitations with existing approaches.

This paper describes the initial development of a measure of QoL for PwPSC in the UK, conducted for a doctoral study [11]. The measure was developed broadly in line with the widely-used European Organization for Research and Treatment of Cancer (EORTC) Quality of Life Group (QLG) Guidelines for developing cancer-related QoL modules [12]. QLG “modules” are usually designed for specific cancers, to be used alongside the core EORTC quality of life questionnaire (the QLQ-C30), but the Guidelines have also been used for stand-alone questionnaires, such as the EORTC QLQ-SWB32 [13], which this study aimed to produce.

The QLG Guidelines state that the first phase of development involves identifying QoL issues of importance to patients via (i) a literature review of existing questionnaires, (ii) interviews with patients at all disease stages, and (iii) interviews with healthcare professionals. When this study was initially designed, four categories for severity of PSC were hypothesised. However, after the student (EM) had been appointed to the study, further reflection by the specialist liver clinician (DT) and discussion amongst liver specialist colleagues led to revision of the hypothesised stratification, such that these initial four categories were increased to six. This change derived solely from the in-depth familiarity with PSC-related medical research and practice of these experienced liver specialists.

PSC is a rare condition, and it is generally challenging for research to identify and recruit people with rare diseases [14], especially those whose conditions are more advanced. In the time available for the study, it would have been impossible to identify, still less recruit, sufficient participants in all six categories, when some of those categories were for levels of disease severity which were themselves rare. It was therefore necessary to adapt the research design. We did this by adopting some variations to the EORTC QLG Guidelines which the EORTC QLG already identifies as acceptable, alongside a few other modifications.

In recent years, online resources such as social media, blogs, and online surveys have been used to explore and collect information about the lived experiences of patients [15, 16]. There is evidence to suggest that these methods are valuable for identifying a broad range of relevant issues, and can identify sensitive issues that people may feel uncomfortable discussing face-to-face with researchers [14, 17–19]. For example, in comparing three methods for eliciting PRO concepts for ankylosing spondylitis, Humphrey et al. [17] derived a greater range of symptom concepts via a social media review of patient chatrooms than in one-to-one interviews, although the chatroom data had less depth of understanding than the interview data. Developing questionnaires using standard methods incurs a heavy cost on resources [17, 18]. Newer approaches using secondary sources to explore the lived experience of a condition potentially offer a resource-efficient way of identifying issues of relevance to patients, which are less burdensome than usual qualitative research approaches [17]. This is particularly important in the field of rare disease, where recruitment is challenging, funding resources tend to be limited, and usual methods are not always possible to follow [20]. The use of mixed methods is also recommended, as it maximises the utility of data collected from small samples [17].

In the modified first stage of our study, we therefore initially identified potentially relevant QoL issues for PSC from secondary sources and then explored these in discussion with PwPSC and expert clinicians. This paper describes this modified approach, and considers related benefits and limitations.

## Methods

We broadly followed the EORTC QLG Guidelines [21] with some variations and modifications, in particular with regard to using in-depth individual interviews with people with PSC to identify issues. For this study, we identified potentially relevant QoL issues from: (a) responses to an existing survey conducted by the charity PSC Support, and (b) from validated QoL questionnaires developed for conditions with some similarities to PSC. We then explored these issues in discussion, first with PwPSC, and then with experienced clinicians, either individually or in groups, depending on participants’ availability and wishes.

### Procedures

#### Issue identification

##### PSC Support survey

We extracted QoL related issues from an online survey with people who self-reported themselves as having PSC (it was not possible to verify respondents' clinical status), run by the charity PSC Support in 2014–2017. The survey, developed by PwPSC, comprised nine questions, covering the lived experience of PSC and opinions about potential impacts of new treatments. To identify relevant QoL issues, we analysed all survey respondents’ answers to one question which asked them to “*describe the most difficult part of living with PSC*”. We were seeking to obtain as wide and exhaustive a set of issues as possible, for exploration with participants in our empirical research. We therefore extracted all issues we identified from every response to this question. We did not set any limit on the numbers of themes from any single response, because for this study we were interested in the breadth of issues, not how many issues any one individual survey participant might identify.

##### Literature review of validated questionnaires

We searched the literature for QoL questionnaires which were likely to include issues of relevance to PwPSC, either through being validated for use with people with conditions often co-morbid with PSC or with similar clinical features to PSC. Seven such conditions were identified through discussions with two experienced liver consultants: (1) primary biliary cholangitis (PBC), (2) IBD, (3) pancreatitis, (4) liver disease, (5) cholangiocarcinoma and gallbladder cancer, (6) colorectal cancer, (7) hepatobiliary cancer.

We aimed to identify potentially relevant QoL issues, rather than all validated QoL questionnaires for each condition, so we searched for existing systematic reviews of relevant questionnaires. We searched three electronic databases from inception to January 2017 (MEDLINE, PsycINFO, The Cochrane Library) using specific condition terms (e.g. IBD), combined with terms for ‘QoL’ and ‘questionnaire’. We de-duplicated and downloaded identified articles. We searched reference lists from these systematic reviews to identify relevant QoL questionnaires and screened remaining papers against our eligibility criteria (Table 1) for any further questionnaires. We listed all identified questionnaires and extracted: study/participant characteristics, questionnaire items, response options, and QoL issues underlying each item.

**Table 1:** Study eligibility criteria for the literature review of validated questionnaires

Study design	Any study validating a disease-specific questionnaire measuring quality of life, health-related quality of life, or well-being
Population	Adults with a diagnosis of: cholangiocarcinoma, gallbladder cancer, colorectal cancer, hepatocellular carcinoma, inflammatory bowel disease, liver disease, pancreatitis
Outcomes	Quality of life issues included in the questionnaire items
Exclusions	• Conference abstracts
	• Studies of questionnaires which focused on a single domain of quality of life (e.g. work productivity or itch)
	• Studies which did not include patients in the development of the questionnaire
	• Non-English language papers
	• Validation of identified tools in further languages
	• Studies which included children and adolescents (< 18 years)

#### Discussions about the issues list

##### People with PSC (PwPSC)

Our primary aim in discussions with PwPSC was to explore the relevance, importance, phrasing, and breadth of coverage of the identified issues (Table 2). We also sought, as a subsidiary aim, to explore the applicability of our hypothesised staging for severity of PSC. We conducted individual or group discussions in-person or via telephone (for PwPSC unable to travel to London), and provided participants with the issues list in paper format.

**Table 2:** Aims of discussions with PwPSC and clinicians, and data sources for assessing each aim

Aim	Data source
Relevance and importance of issues	Quantitative responses on issues list
	Qualitative responses regarding issues selected
Phrasing of issues	Qualitative responses regarding phrasing and preferences
	Qualitative responses regarding whether issues were appropriate (e.g. anything upsetting)
Breadth of coverage	Qualitative responses regarding whether any issues were missing

We developed a structured schedule to guide the discussions, adapted from the QLG guidelines [12], which comprised five steps: (1) participants described one or two of the most important issues for their QoL, (2) participants rated the relevance (Yes/No) of each issue on the issues list, and from these, selected the most important, (3) participants discussed their selections, (4) participants discussed issue phrasing, including the appropriateness of language used, (5) participants stated whether any important issues for their QoL were not listed (see Additional file 1). Participants were not asked about the hypothesised PSC staging.

All participants chose a pseudonym to use during the discussions (used in the example quotes in the results section). In-person group discussions were facilitated by EM and observed by BV. Individual interviews and online group discussions were conducted by EM alone. BV is an experienced qualitative researcher; EM was trained in qualitative data collection as part of her doctoral studies. Prior to the empirical work with participants with PSC we ran a series of trial group discussions, facilitated by EM, on which BV fed back, so as to develop EM’s skills and revise and fine-tune the discussion process and guide.

Ethical approval was granted by the West London & GTAC Research Ethics Committee in August 2017 (reference number: 17/LO/1108).

##### Clinicians

After completing all the research discussions with our participants with PSC, we conducted group and individual discussions with clinicians, either in-person or by telephone, following the same interview schedule as with PwPSC, and all run by EM alone. We used a reduced issues list for these discussions, which listed only those issues which ≥ 60% PwPSC had marked as relevant, since the tool we sought to develop was to be patient-centred and should therefore prioritise items of most relevance to PwPSC. The 60% cut-off is a standard EORTC QLG decision rule for issue reduction [12].

### Recruitment

#### PwPSC

We hypothesised six categories for severity of PSC: (1) PSC only, (2) PSC with IBD, (3) awaiting liver transplant, (4) post-liver transplant, (5) recurrent PSC post-transplant, (6) experience of a related cancer. We recruited PwPSC via PSC Support and from an NHS clinic at the Royal Free Hospital, London (Nov 2017-Mar 2018). MW (Chair of PSC Support) invited PSC Support members via email and the charity website. We purposively sampled potential participants, aiming to recruit a minimum of six in each category. PwPSC were suitable for inclusion if they were: ≥ 18 years old, a UK resident, and able to speak English to a sufficient level for the discussions. PSC-related cancers comprised: cholangiocarcinoma, gallbladder cancer, hepatocellular carcinoma, and colorectal cancer. To identify patients with more advanced disease, the lead clinician (DT) contacted patients who were awaiting a liver transplant, or had experience of cancer, via email. We screened potential participants by telephone, collecting basic demographic and clinical information to assign PwPSC to one of the six PSC categories. We also asked PwPSC to rate the severity of their PSC as mild, moderate, or severe.

#### Clinicians

We aimed to recruit 10–12 physicians and nurses with experience of caring for PwPSC in the UK. Few clinicians in the UK have experience with PSC, so we used a snowballing technique to identify and recruit clinician participants [22]. Potentially suitable clinicians were sent the study information sheet and consent form via email and invited to respond if interested.

### Data analysis

#### Constructing the issues list

We analysed the PSC Support survey responses to the “difficult” question thematically and inductively, coding at the semantic level [23], and keeping the wording close to the participants’ original words. We organised issues from the literature review according to the themes identified, and removed duplicates. We developed a set of decision rules to reduce the issues list systematically, based on existing literature for item reduction [24–26] (Table 3), so that the list for the discussions with PwPSC would be as manageable as possible. We applied these decision rules after we had collected all the issues together; that is, independently of the original sources.

**Table 3:** Stage 1 decision rules for excluding issues from the full issues list

Decision rule	Description	Examples
1. Overlapping or overly specific [1]	Where multiple issues covered the same concept, issues which were overly specific were excluded. This reduced the number of included issues without excluding the underlying concept from the issue list	‘*Frequent urination at night’* excluded as covered by ‘frequent urination’
		‘*Ability to get all work jobs done*’ excluded as covered by ‘ability to work’
2. Double-barrelled issue [2, 3]	Any issue made up of two concepts contingent on one another were excluded. These issues ask two separate questions and are therefore difficult to respond to because a single response option may not be suitable	‘*I feel frustrated that I can't go out and enjoy myself*’
		‘*I feel so tired I have to go to bed early*’
3. Phrased in a colloquial manner [2, 3]	Issues using colloquial language were excluded. The use of colloquialisms or common idioms is problematic because they may not be understood uniformly and the literal meaning may differ from the intended meaning	*‘Feeling off my food’*
		*‘Feeling worn out’*
		*‘My future seems dark’*
4. Phrased in a complex manner [2]	Issues phrased in a complex manner were excluded, because they require a high level of cognitive skill to comprehend, and risk being misinterpreted	‘*My daily activities are hampered by concerns about the impact of my condition on my family situation*’

Our decision rules were that we excluded issues if they were: (1) overlapping or overly specific [24], (2) double-barrelled [25, 26], (3) phrased in a colloquial manner [25, 26] or (4) phrased in a complex manner [25]. We were cautious not to exclude any issues at this stage, so if in any doubt we included issues we considered possible duplicates, and ensured that in those cases we asked the empirical research participants specific questions about those particular issues, including which they preferred, for example “feeling fatigued” or “feeling tired”. We also ensured that excluding double-barrelled or complex or colloquial terms did not mean that the underlying concepts were excluded, by checking that included issues covered these concepts, for example feeling fatigued or tired is equivalent to feeling “worn out” (Table 3). Three authors (EM, BV, MW) screened the issues list and excluded issues based on these decision rules. Any differences were discussed until a consensus was reached.

### Exploring participants’ responses to the issues list

We calculated participants’ relevance and importance scores for each issue. Audio recordings were transcribed by a professional transcription service, and we checked and reviewed transcripts and participants’ written responses for any new issues raised during the discussions. We considered new issues for inclusion if they were suggested by at least two participants or deemed to be highly important [12]. We explored participants’ understanding of issue phrasing and the appropriateness of language used. As a post-hoc analysis, we also explored the appropriateness of the hypothesised PSC categories.

## Results

### Issue identification

#### PSC Support survey

There were 445 respondents to the PSC Support survey, and 301 of these replied to the “difficult” question. The length of their responses varied, with 43% (130/301) describing just one or two issues (e.g. “extreme fatigue and poor memory”). The other 171 respondents gave longer answers with more detail on their experiences, which therefore led to the identification of more themes than from the brief responses. All issues contained in each response, however long, were identified separately. So, for the example of the response above, fatigue and memory were separated, because they are two distinct issues, although both provided in a single answer from an individual respondent.

**Fig. 1:**
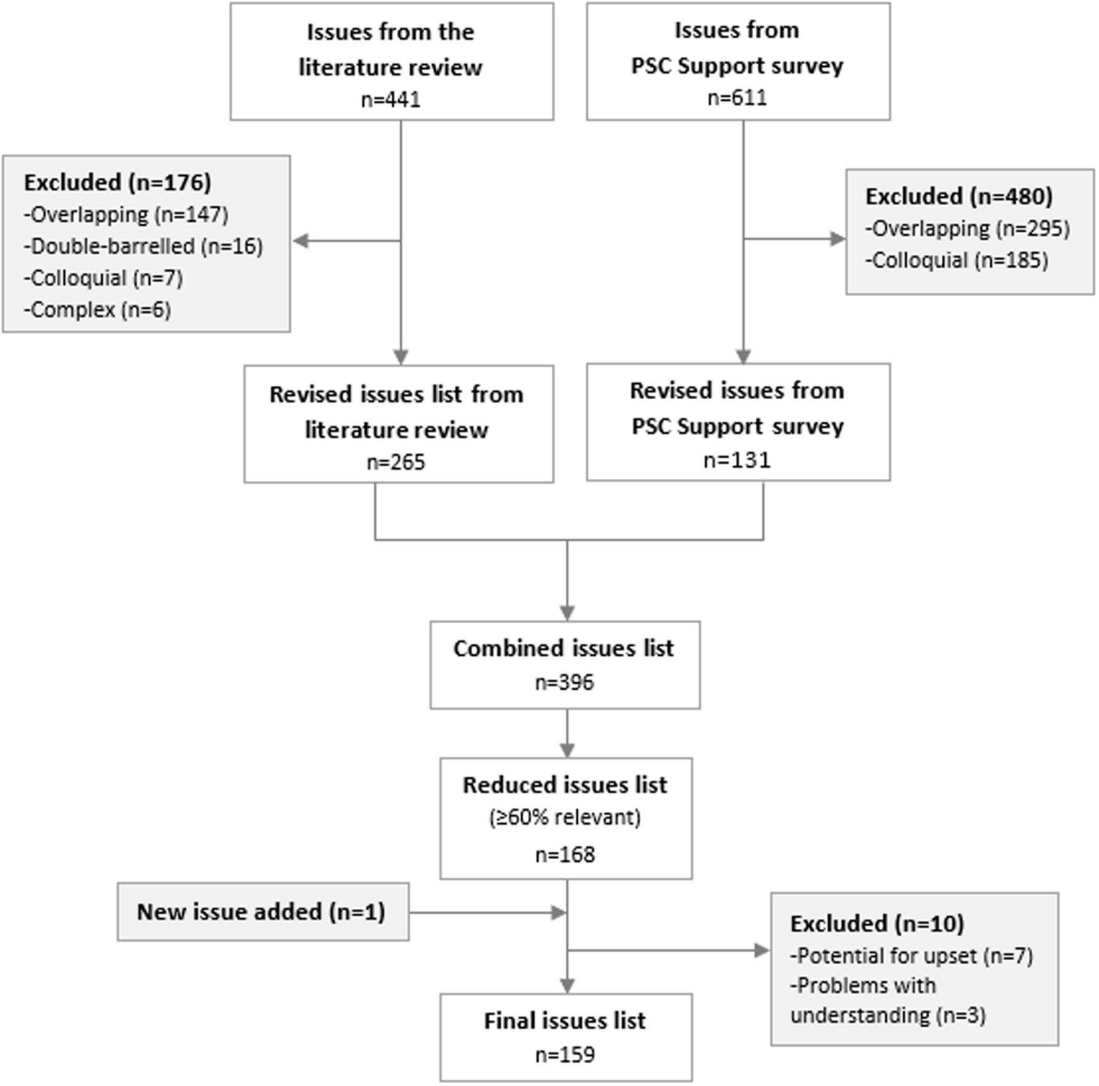
Retention and exclusion of issues identified from the PSC Support survey and the literature review of existing QoL tools

From all the answers we extracted 611 unique issues and derived eight themes: (1) physical health (n = 170), (2) emotional health (n = 97), (3) cognitive health (n = 11), (4) social functioning (n = 85), (5) close and intimate relationships (n = 39), (6) uncertainty, knowledge, and information (n = 117), (7) work, education, and finances (n = 43), and (8) experience of care and treatment (n = 49) (Table 4, column 2). We excluded 480 issues based on the decision rules: overlapping or overly specific (n = 295), colloquial language (n = 185) (Fig. 1).

**Table 4:** Number of issues by category for each step in the study

Categories	PSC support survey	Literature review	Combined (Stage 1 issues list)	60% Relevant
Overall QoL	–	4	3	–
Physical health	170	148	144	45
Emotional health	97	87	78	30
Cognitive health	11	19	7	4
Social functioning	85	68	46	18
Close relationships	37	–	33	22
Sex life	2	23	–	–
Diet	–	17	–	–
Body image	–	10	–	–
Information and uncertainty	117	–	35	30
Work, money, education	43	20	17	10
Experience of care and treatment	49	27	33	9
Spirituality	–	18	–	–
**Total**	**611**	**441**	**396**	**168**

#### Literature review of validated questionnaires

We identified 4,086 articles, excluded 4,030 based on title/abstract, and conducted a full-text appraisal for 56 studies (describing 39 questionnaires). Twenty-three questionnaires met the eligibility criteria (see Additional file 1). From these, we extracted 441 unique issues and organised them according to 11 domains (based on domains from the included questionnaires): (1) overall QoL (n = 4), (2) physical health (n = 148), (3) cognitive health (n = 19), (4) emotional health (n = 87), (5) diet (n = 17), (6) body image (n = 10), (7) sex life (n = 23), (8) social life (n = 68), (9) employment and finances (n = 20), (10) medical care and treatment (n = 27), and (11) spirituality (n = 18) (Table 4, column 3). Based on the decision rules, we excluded 176 issues: overlapping or overly specific (n = 147), double-barrelled (n = 16), colloquial language (n = 7), and complex phrasing (n = 6) (Fig. 1).

We combined all the issues from the survey and the literature review to form a single issues list, sorted into broad categories for type of issue and by themes identified from the PSC Support survey data. This list was too long to be covered completely in each session, so we changed the order of the list between discussions to ensure all issues were discussed at least once. We asked participants to complete outstanding issues at the end of the session or at home and provided them with a freepost return envelope.

### Discussions about the issues list

#### PwPSC

##### Recruiting PwPSC and categorising the condition

Forty-nine PwPSC responded to the study invitation, and we screened 47 (two PwPSC did not ever respond again after their first response, despite two email reminders). We included 28 PwPSC and grouped them into the six hypothesised PSC categories (Table 5); 23 participants were recruited via PSC Support and five via the Royal Free Hospital.

**Table 5:** Number of people with PSC who took part in Stage 1 discussions according to PSC category and severity

Category	N	Mild	Moderate	Severe
(1) PSC only	6	3	2	1
(2) PSC and inflammatory bowel disease (IBD)	9	2	7	0
(3) Assessed or waiting for a liver transplant	3	0	0	3
(4) Post-liver transplant	5	5	0	0
(5) Recurrent PSC post liver-transplant	3	0	3	0
(6) Experience of a PSC-related cancer	2	2	0	0
**Total**	**28**	**12**	**12**	**4**

Participants were selected primarily based on their PSC category. We wanted to ensure numbers in categories were as balanced as possible, so once a sufficient number of people in a category had participated, no more people in that category were invited to take part. We recruited our minimum target numbers of six participants to categories (1) and (2) (exceeding the target for category 2, because more people in this category offered, and we did not want to turn down people who had already agreed), and just under (five participants) to category (4). For categories (3), (5) and (6), which are rare occurrences for an already rare disease, we recruited three, three, and two participants respectively. Of the 47 people who were screened, 19 did not take part in the discussions (see Table 6 for the reasons).

**Table 6:** Reasons why 19 participants who were screened did not take part in the discussions

Reason	N
Unable to travel, and refused phone participation	7
Already filled PSC categories	6
No time	3
Changed mind	3

We held nine group discussions (five via teleconference and four at UCL), and nine individual telephone discussions. One participant did not return their outstanding issues list pages, because during the discussion they received an urgent call for hospital admission, and we were unable to give instruction about how to complete and return the document. We followed up three weeks later, but received no response, and, because we had no way of knowing the participant’s health status, considered that it was inappropriate to pursue this further.

##### Participant characteristics

Participants were mostly male (61%), white British (82%), and married (61%) (Table 7). Median age was 50 years (range = 22–70). The majority had experienced symptoms for > 10 years (n = 14) or 6–10 years (n = 9). Twelve participants (43%) rated their PSC as mild, 12 (43%) rated it moderate and four severe (14%). Twenty-four participants (86%) had a co-morbid condition, most commonly, IBD (18/28), osteoporosis (6/28) or other autoimmune conditions (5/28).

**Table 7:** Characteristics of PSC participants

Participant characteristic	N (%)
Gender (male)	17 (61%)
Age (median = 50; range = 22–70)	
20–29	1 (4%)
30–39	6 (21%)
40–49	5 (18%)
50–59	9 (32%)
60–70	6 (21%)
Ethnicity	
White British	23 (82%)
White other	3 (11%)
Asian British	2 (7%)
Marital status	
Single	7 (25%)
Married	17 (61%)
Divorced	4 (14%)
Time since first symptoms	
> 10 years	14 (50%)
6–10 years	9 (32%)
1–5 years	3 (11%)
< 1 year	2 (7%)
Co-morbidities	
IBD	18 (64%)
Osteoporosis	6 (21%)
Other autoimmune conditions^a^	5 (18%)
Asthma	2 (7%)
Depression	2 (7%)

^a^Ankylosing spondylitis, psoriasis, pulmonary sarcoidosis, rheumatoid arthritis

##### Relevance and importance scores

At least two participants marked every issue as relevant. 60% or more participants marked 168/396 issues as relevant, and 30/396 issues as important (Table 8).

**Table 8:** Issues marked as important by ≥ 60% PSC participants

Categories	Issues
Overall QoL	Impact of PSC on quality of life
Physical health	Feeling unwell
	Never feeling 100%
	Feeling exhausted, physically and/or mentally
	Itch
	Itching through the night
	Jaundice
	Cholangitis flare-ups
Emotional health	Ability to enjoy life
	Feeling worried about PSC
	Concerned about being a burden on others
	Concerned about eventual death due to PSC
Cognitive health	Ability to concentrate
Social functioning	Ability to do usual activities
	Ability to lead a normal life
	Impact of PSC on social life
Close relationships	Impact of PSC on family life
	Impact of PSC on partner
	People thinking PSC is due to alcohol
Information and uncertainty	Uncertainty about the impact of PSC on life
	Uncertainty about the impact of PSC on my family
	Uncertainty about when or if I will need a liver transplant
	Concerned about how PSC will affect the future
	Concerned about cholangitis attacks
	Concerned about liver transplant
	The variability of symptoms
Work, money, education	Ability to work
	Losing confidence about being able to do a job full-time
	Impact of PSC on financial situation
Experience of care and treatment	Unplanned hospital visits

##### Breadth of coverage

Nine PSC participants suggested 12 additional issues they felt were missing from the issues list. One issue, suggested by two participants, was deemed very important, and was added to the issues list: ‘concerns about developing cancer’. The other 11 issues suggested by a single participant were not included as they overlapped existing issues (e.g. ‘sleep reversal’ covered by ‘difficulty sleeping’), or were deemed to be overly specific (‘difficulty claiming benefits’).

##### Phrasing of issues

Some participants with PSC identified similarities between issues in the ‘physical health’, ‘cognitive health’, ‘close relationships’, and ‘uncertainty, knowledge and information’ themes. In some cases, there was a clear consensus among participants about the suitability of phrasing, for example, ‘feeling exhausted, physically and/or mentally’, was preferred by most participants among the fatigue related issues because it was easily understood, covered the severity of their fatigue, and highlighted how fatigue could be a cognitive symptom as well as a physical one. In some cases, however, participants found these decisions difficult to make. For example, when asked about the issues ‘disturbed sleep’ and ‘disrupted sleep’, one participant commented:

I agree with Anna [*other group participant*], really hard to choose between the two because they are so similar. You wake up itching and it's… I suppose I put disruptive just because it disrupts your sleep pattern … I just think they're very similar and I couldn't pick between the two, I just went for disruptive.—**Louise (post-liver transplant, mild, age 35, 7 years since diagnosis)**

Participants with PSC described 12 issues as being potentially upsetting, mostly those describing strong negative emotions (e.g. ‘feeling like I've been handed a death sentence’). Despite their sensitive nature, however, all participants felt that these issues should be retained, because they found that they frequently experienced these issues, and felt that it was important to assess them. For example, one participant said:

No, I mean, I deal with these things on a daily basis so, you know, seeing them written down can be upsetting, they can take you aback a little, but the reality of it is that it’s there all the time. It’s my life, so… But I don’t think it should be shied away from.—**Adam (recurrent PSC, moderate, age 22, 7 years since diagnosis)**

Our participants described problems understanding seven issues, due to ambiguity or difficulty with interpretation. For example, even though diagnosed with “Primary Sclerosing Cholangitis”, some participants still did not understand the term ‘cholangitis flare-ups’. There was also some variation in how participants interpreted the issue ‘the variability of symptoms’; some thought it meant that PSC symptoms vary between people, whilst others understood it on an individual level, as meaning that an individual with PSC can experience a wide range of symptoms.

#### Clinicians

##### Participant characteristics

Eleven clinicians (six physicians and five nurses) took part in three face-to-face group discussions and three individual telephone discussions (Table 9). These participants had worked with PwPSC for between four and 40 years (median = 18 years).

**Table 9:** Characteristics of clinician participants

Participant characteristic	N
Gender (male/female)	5/6
Age	
35–44	5
45–54	4
55+	2
Job title	
Consultant	4
Clinical research fellow	1
Specialist registrar	1
Clinical nurse specialist	2
Transplant co-ordinator	2
Research nurse	1
Years qualified	6–43 (median = 21)
Years working with PSC patients	4–40 (median = 18)

##### Relevance and importance scores

60% or more clinician participants marked 121/168 issues of the reduced issues list as relevant (Table 4, column 4) and 46/168 as important. Every issue was marked as relevant by at least seven of these participants, and as important by at least two.

##### Breadth of coverage

Seven clinician participants suggested 10 issues were missing from the issues list. Five of these had been included in the initial full issues list but, because less than 60% of our PwPSC participants had marked them as relevant, they were not included in the list presented to clinician participants. We added one of five new issues clinician participants suggested—concern about developing cancer—to the list, because it had also been mentioned by PwPSC participants. We did not consider there were any strong reasons for including the other four issues, which were either overly specific (e.g. difficulty obtaining a mortgage) or covered by another issue (e.g. ‘concern about developing cancer’ covered ‘uncertainty about cancer’).

##### Phrasing of issues

The main issues clinician participants reported as similar described: general health perceptions (‘feeling ill’ and ‘feeling unwell’); specific symptoms (‘muscle aches’ and ‘aches in limbs’); coping with condition (‘I have accepted my condition’ and ‘I have learned to live with PSC’); anxiety (‘feeling worried about PSC’ and ‘feeling stressed’); and work life (‘ability to work’, ‘impact of PSC on work’). These participants said that the term ‘cholangitis’ may cause problems with understanding as it is a “technical term”, and that understanding would depend on “how experienced your patient is”. Five issues from the ‘coping with condition’ section of the issues list (e.g. knowing PSC is shortening my life) were described as potentially upsetting or distressing for PwPSC, and clinicians suggested they be rephrased more sensitively. For example, one clinician participant said:

I do think, feeling that my life may be limited, is a distressing question. … And that there might be people earlier on in the illness who haven’t thought about that as much and it might distress them. Having said that I think they’re quite important factors in quality of life. … a question about not being 100% sure about how the condition will pan out, might be a way of saying it in a less distressing way.—**Sophie (Clinical research fellow, 4 years working with PSC patients)**

### Revisions to the issues list

In all, PwPSC and clinician participants suggested 17 new issues, of which we included one, and identified problems with the phrasing of 19 issues, due to the potential for upset (n = 12) or problems with understanding (n = 7). Of these we excluded 10 issues and re-phrased nine.

The final outcome from this first phase was a list of 159 issues agreed by all participants as candidates for items for inclusion in a provisional measure.

### Hypothesised PSC categories

During discussions with our PSC participants we found a distinction in the experiences of people in categories 1 (PSC only) and 2 (PSC and IBD). People in both groups differed in their experience of symptoms. Some were asymptomatic (or experienced few symptoms), while others experienced regular symptoms, which they characterised as moderate to severe (Table 5). We therefore determined that it would be more meaningful to divide each of these categories into two: *mild,* and *moderate-severe*, thus revising our categories for severity of PSC to eight rather than six.

## Discussion

### Main findings

In order to address the challenge of stratifying this rare disease for severity, and related difficulties with identifying and recruiting participants to this study, we modified the standard methodology for deriving QoL issues, albeit with some alignment with variations which the EORTC QLG considers acceptable [12]. We merged two stages of development which are usually conducted consecutively: open-ended interviewing and discussion of a provisional issues list. We initially identified a large number of unique QoL issues from the survey (n = 611) and literature review (n = 441). We reduced these via our decision rules, but the final issues list for exploring with PwPSC and clinician participants still contained a large number of issues (n = 396). At least two participants with PSC considered every issue in this long list relevant, and at least seven of the 11 clinician participants considered all issues from the reduced list relevant. More than two-fifths of the issues resonated with our PSC participants: over 60% of them marked 168 of the 396 issues as relevant. PSC and clinician participants suggested seventeen new issues. However, most of these were similar to existing issues, and we added only one of them to the issues list. In discussions, participants described problems with the phrasing of 19 issues: 12 as potentially upsetting, and potential problems with understanding seven. Both groups, however, said that the ‘upsetting’ issues should be retained, although clinician participants suggested they might be rephrased to be more sensitive.

The outcome was a set of 159 issues which all participants agreed were the priority to go forward for possible inclusion in the provisional measure. This list of 159 issues had been reduced from the 396 issues discussed with participants, themselves selected from an initial set of 1052 issues identified from the PSC Support survey and our review of existing questionnaires.

Following input from PSC participants, we concluded that there should be eight categories for severity of disease, rather than the four, and later six, categories hypothesised at the outset of the study.

### Strengths and limitations

A strength of this study was the recruitment of PSC participants living with varying stages and levels of disease severity. This enabled the study to obtain a broad range of perspectives, and also facilitated deeper reflection and development of categorisation for the currently ill-defined stages of PSC severity. We began the group discussions with broad open questions so that participants could raise QoL issues unprompted, and facilitated participants’ sharing of their individual experiences and thoughts with one another. This was helpful for building and developing an understanding of the lived experience of PSC. The issues list was a useful catalyst for PSC participants to discuss different impacts of the condition. For example, some participants said they might not have discussed certain areas of the issues list unprompted, such as issues about mental health. The wealth of quantitative and qualitative data we collected informed our decisions about the retention of issues and optimised the use of participants’ time [20]. Telephone interviews with PwPSC who were unable to travel to our offices, due to distance or health problems, enabled their participation. Only nine of the 28 participants who took part were able to attend in person, although the study provided cover for any travel cost they might incur.

Overall, using secondary sources to initially derive QoL issues was less demanding for PSC participants. Using data from the PSC Support survey presented descriptions of illness from the patient perspective, and thereby provided insight into the main difficulties PwPSC encounter with their condition. Our further exploration of these in group discussions with PSC participants, enabled sharing of experiences, and thereby further and deeper understanding of living with the condition. Participants with PSC understood most issues, which may have been in part because the literature review allowed us to identify issues from validated questionnaires, the wording of which has already been pilot-tested with patients, and some assessed for translatability [26].

In common with Humphreys et al. [18], our approach was limited in obtaining an in-depth understanding of the whole lived experience of PSC. We began interviews with open questions, and explored themes from the issues list with PwPSC participants, but it was not possible to cover all areas thoroughly in the available time. This contrasts with typical individual interviews which allow one to probe responses in more depth, including how frequent, severe, or burdensome symptoms and impacts of illness may be for patients.

We were aware of the challenges in recruiting participants with a rare disease, and therefore used two recruitment strategies for this study: via PSC Support, and from an NHS clinic. Despite these strategies, and despite enabling remote participation, we under-recruited to four of our six hypothesised categories for PSC severity.

Clinical data for most of our PSC participants were limited, because they were recruited via PSC Support, rather than the hospital clinic. Any clinical data for these participants were therefore based on participants’ self-report, and we were unable to verify reported diagnoses or check recent blood test results. Ratings of PSC severity were also according to participants’ recent subjective experiences of the condition, as opposed to any clinical indications of early or progressed disease.

### Implications and future research

Our findings suggest that there is considerable value in using secondary sources other than pre-existing questionnaires to identify issues of relevance and importance to patients with rare conditions. However, it is important that these data are considered alongside data generated from primary sources. At present little research compares differing methodologies for issue identification [17, 19], and it is therefore unclear whether questionnaires developed using distinct sources and methodologies vary in any meaningful way.

A key challenge for this study was the lack of consensus about PSC staging. Our discussions with PSC participants helped add detail to the limited scientific understanding about the natural history of PSC and resulted in the expansion of our categories for disease severity, already revised from four to six, to a set of eight. Further qualitative research with PwPSC across our eight newly-hypothesised categories is crucial for deepening this understanding. Future research may also benefit from exploring associations between the lived experience of PSC and measures of disease progression, such as the Enhanced Liver Fibrosis (ELF) test [27], once they have been shown to be reliable and valid.

## Conclusion

In light of the heavy resource burden associated with the development of questionnaires [18], and the particular challenge for developing questionnaires for people with rare conditions, our study was successful in identifying a broad range of relevant issues for PwPSC. More individual, in-depth interviews would have enabled a greater depth of understanding, but it is unclear whether this would have made any meaningful difference to the issues identified. Our study suggests that there is value in employing non-standard methods for developing QoL questionnaires, particularly for rare conditions which are poorly understood and/or under-researched. The next stage of this study used findings from this stage to develop and pilot-test a QoL questionnaire following standard guidelines. A subsequent paper will report on that work.

## Supplementary Information

**Additional file 1.** **Supplementary files.** (1) Individual/group discussion schedule. (2) Validated questionnaires included in the literature review.

## Data Availability

The datasets analysed during the current study are available from the corresponding author on reasonable request.
