# Supplementary material for: Quality of life (QoL) for people with primary sclerosing cholangitis (PSC): a pragmatic strategy for identifying relevant QoL issues for rare disease
Source: J Patient Rep Outcomes. 2022 Jul 15;6:76. doi: 10.1186/s41687-022-00484-5 (PMC9287498; doi:10.1186/s41687-022-00484-5)
Supplement: Supplementary file 1 — Additional file 1. Supplementary files. (1) Individual/group discussion schedule. (2) Validated questionnaires included in the literature review. [file 41687_2022_484_MOESM1_ESM.pdf]

Supplementary files

(1) Individual/group discussion schedule

A. Introductions **(Time: 00 to 00+13)**

Welcome everyone and thank you for taking the time to come to this group discussion where we will be looking at quality of life issues for people with PSC. My name is EM and I am a PhD student here at UCL and I will be running the group today.

The session today will be **audio-recorded** to ensure we capture everything that is said. To help with the recordings please can you ensure you **respect** other participants during the session and make sure you wait for people to finish speaking before you begin. This is BV, she will mostly not be involved in the group discussion, but will instead observe interactions and take notes, partly as back-up in case of a recorder malfunction.

Today's session will be kept strictly **confidential**. Please respect this by keeping everything said today within these four walls. To ensure confidentiality we would like you to use **pseudonyms**, or fake names, when speaking to each other. Before we begin please could you look over the **participant information sheet** and sign the **consent form** using your initials in the boxes. Once signed I will photocopy each consent form so that we both have a copy. Whilst I do this, please **pick a pseudonym** and write it on the place card in front of you.

We have **tea, coffee and water** here, so please help yourself at any point during the session, and if you need to take a **break** you can leave the room, you don't need to say anything. **Toilets** are available if you walk back out of the entrance, and carry on straight past the lifts. Finally, if you could put your **phones** on silent, or switch them off, that would be great.

Aim of today **(Time: 00+13 to 00+15)**

Our overall aim for this project is to create a questionnaire that measures quality of life for people living with PSC. To start this process we have done some research and put together a list of issues or problems which may affect the day to day life of people with PSC. These can be things like symptoms (for example, pain), feelings (such as feeling sad) or any impacts these things can have on a person's day to day life (for example, a person's ability to work).

This list we have put together has come from two different sources. Firstly, we looked at a survey which was run by PSC Support a couple of years ago and which asked people with PSC to describe the most difficult part of living with their condition. Secondly, we looked at a range of existing questionnaires which have been developed for conditions which are in some ways quite similar to PSC.

What we've ended up with is quite a long list of issues, over 300 to be precise. We cannot include all these issues in a questionnaire, because it would take far too long to complete, so what we would like you to do today is to go through this list and select which of these issues you think are the most important ones to include in a questionnaire about quality of life.

B. Group discussion

1. Starting the discussion **(00+15 to 00+30)**

Before we look at the list of issues, could we start by spending the **first ten minutes** or so going round the group, with everyone saying what the **most important issue** for living with PSC is for them?

[*Turn to the person on the right and ask them to start off*]

- Please could you state your pseudonym and then describe one or two of the most important issues for your quality of life?

[*Briefly summarise the person's issues and move to the next person*]

If respondent is commenting at length:

- Thanks very much, so you're saying [****] is the most important issue for you? We can come back to this later when we look through the issues list.

[*After everyone has spoken, summarise issues identified*]

**2. Presenting the issues list**	**(Time: 00+30 to end)**

We’re now going to move on to the list of issues.

[*Hand the issues lists around the group*]

Please could you add today’s **date** and your **pseudonym** to the top of the list? We’ve divided the list into smaller, more manageable chunks according to the type of issues described. This is just to help us navigate through the list during the session. As I’ve said before this list is very long, so it’s quite likely that we won’t be able to get through the whole thing today. If this happens you can still finish the questionnaire at home, if you wish to, and return it to us in the post using a pre-paid envelope.

Before we look at the list, I’ve got a few copies of an existing questionnaire developed for people with cancer for you to look at. This should help you get a rough idea of what we’re trying to achieve by the end of the whole project.

[*Hand out the FACT-C*]

As you can see there are positively worded statements, such as ‘*I get emotional support from my family’* as well as negatively worded statements such as ‘*I have a lack of energy’*. This will also be the case in our list of issues. So if you think something like ‘getting emotional support from family’ is an important thing to include in a quality of life questionnaire you would mark this as important on the list. At the bottom of this page there is a statement about a person’s sex life. We too have a section in our list of issues which covers intimate relationships and sexual functioning. We understand that some people may not feel comfortable discussing these issues in a group, so we’ve added this as an optional section to discuss at the end of the session. If you’d rather not discuss this in a group, that’s fine, you can fill this out on your own at the end of the session or complete it at home if you wish to.

When working on the list of issues today, to make the session slightly easier, we will be working through the list **one page at a time**. When looking at a page we’d like you to start by adding a tick in the ‘relevant column’ for those issues you think would be relevant to include in a quality of life questionnaire. You do not need to spend too much time thinking about this, as there are no wrong or right answers.

Once you’ve completed the relevant column we’d like you to move on to the important column. In this column we’d like you to spend a little more time thinking about which of the issues that you marked as relevant are the most important ones to be included in a quality of life questionnaire. When doing this keep in mind that we can’t include everything in a questionnaire because it needs to be manageable, so really think about what is most important.

When looking through the list you might notice that some of the issues are quite **similar**. If you mark an issue as important and notice that it is worded in several similar ways, we are really interested in knowing which wording you prefer.

We’d like you to remember that some of the issues have come from questionnaires developed for other conditions, so we’re not necessarily saying that all the listed issues will happen to people with PSC.

[*Move through list page by page*]

Starting with [*first chosen category*] on page 2:

- Please could you read through the list and start by ticking in the **‘Relevant’ column only**, which of these issues you think are relevant to be included in a quality of life tool and which you think are not?
- Once you have completed the ‘Relevant’ column, for the issues you have marked as relevant, could you spend a bit more time thinking **which of these you think are most important** to be included in a quality of life tool?
- If you finish before everyone else, you can move on to the next page. Once everyone has finished we will discuss this page only.

[*Once the group have completed the page, turn to the person on the right and ask them to start off*]

- Could you tell us which of these issues you have marked as important, stating the listed number as well as the issue to help guide us?
- Do you think any of the issues listed are upsetting?

3. Prompts and follow-up questions [*as needed*]

- *If participants pick similarly worded issues*:
  - [X] has mentioned this issue as important whereas [Y] has picked this issue. To me these look like quite similar issues, so could you explain a little more why you selected that particular issue?

- *If the answer is incomplete:*
  - Would you tell me a little more about that?

- *If a given answer is too general and indefinite*:
- In what way? Just how do you mean? Can you give me an example?

[*Repeat for each section*]

D. Wrapping up

We don't have much more time left in this session and need to start coming to the end.

- Can you think of anything else that you have experienced which we haven't discussed?
- Do you have any other comments regarding your quality of life?
- Any further questions?

Thank you all for taking the time to share your views and experiences with us today.

[*If an in-person discussion*] You are welcome to complete your issues list now, in your own time. Or if you prefer, you can take the issues list home with you, complete it there, and then return it to us using this Freepost envelope.
[*If a telephone discussion*] Please could you complete the rest of the issues list in your own time at home, and then return it to us using the Freepost envelope provided.

Please **feel free to contact us** with any further questions or if you would like any updates on the study. If you indicated that you are happy to be contacted in the future on the consent form, we will **keep your personal details safe** and contact you when we start recruiting for Stage 2 or when we have a report of the study. If you said you do not want us to contact you, but change your mind about this, please let us know.

(2) Validated questionnaires included in the literature review

Study ID	Questionnaire name
Colorectal cancer	
Colwell 2010[1]	Functional Assessment of Cancer Therapy/National Comprehensive Cancer Network Colorectal Cancer Symptom Index (FCSI-9)
Rinaldis 2009[2]	Coping with Colorectal Cancer measure (CCRC)
Sprangers 1999[3]<br>Whistance 2009[4]	EORTC* Questionnaire Module for Colorectal Cancer (QLQ-CR38/QLQ-CR29)
Ward 1999[5]	Functional Assessment of Cancer Therapy-Colorectal (FACT-C)
Inflammatory bowel disease	
Alrubaiy 2015[6]	Crohn's and Ulcerative Colitis Questionnaire (CUCQ 32)
Alcala 2004[7]	Shortened Questionnaire on Quality of Life for Inflammatory Bowel Disease (IBDQ-9)
Allen 2013[8]	Patient-reported Disability Measurement Tool for Inflammatory Bowel Disease
Cheung 2000[9]	British Version of the Quality of Life Questionnaire for Inflammatory Bowel Disease (UK-IBDQ)
Drossman 1991[10]	Rating Form of Inflammatory Bowel Disease Patient Concerns (RFIPC)
Guyatt 1989[11]	Inflammatory Bowel Disease Questionnaire (IBDQ)
Irvine 1996[12]	Short Quality of Life Questionnaire for Inflammatory Bowel Disease (SIBDQ)
Peyrin-Biroulet 2012[13]	IBD disability index
Smith 2012[14]	Social Impact of Chronic Conditions–Inflammatory Bowel Disease (SICC-IBD)
Wilcox 2010[15]	Crohn's Disease Burden Questionnaire
Liver-related cancers	
Blazeby 2004[16]	EORTC Questionnaire Module for Hepatocellular Carcinoma (QLQ-HCC18)
Heffernan 2002[17]<br>Yount 2002[18]	Functional Assessment of Cancer Therapy–Hepatobiliary (FACT-Hep)
Kavadas 2003[19]<br>Blazeby 2009[20]	EORTC Questionnaire Module for Liver Metastases from Colorectal Cancer (QLQ-LMC21)
Liver disease	
Anderson 2014[21]	Chronic hepatitis C virus patient-reported outcomes instrument (HCV-PRO)
Gralnek 2000[22]	Liver Disease Quality of Life Questionnaire
Younossi 1999[23]	Chronic Liver Disease Questionnaire
van der Plas 2004[24]	The Liver Disease Symptom Index 2.0
Pancreatitis	
Wassef 2014[25]<br>Wassef 2016[26]	Pancreatitis Quality of Life Instrument

**EORTC* = European Organization for Research and Treatment of Cancer

References

1 Colwell, H. H. *et al.* Psychometric evaluation of the FACT Colorectal Cancer Symptom Index (FCSI-9): reliability, validity, responsiveness, and clinical meaningfulness. *Oncologist* **15**, 308-316, doi:http://dx.doi.org/10.1634/theoncologist.2009-0034 (2010).

2 Rinaldis, M., Pakenham, K. I., Lynch, B. M. & Aitken, J. F. Development, confirmation, and validation of a measure of coping with colorectal cancer: a longitudinal investigation. *Psycho-Oncology* **18**, 624-633, doi:http://dx.doi.org/10.1002/pon.1436 (2009).

3 Sprangers, M. A., te Velde, A. & Aaronson, N. K. The construction and testing of the EORTC colorectal cancer-specific quality of life questionnaire module (QLQ-CR38). European Organization for Research and Treatment of Cancer Study Group on Quality of Life. *European Journal of Cancer* **35**, 238-247 (1999).
4 Whistance, R. N. *et al.* Clinical and psychometric validation of the EORTC QLQ-CR29 questionnaire module to assess health-related quality of life in patients with colorectal cancer. *European Journal of Cancer* **45**, 3017-3026, doi:http://dx.doi.org/10.1016/j.ejca.2009.08.014 (2009).
5 Ward, W. L. *et al.* Reliability and validity of the Functional Assessment of Cancer Therapy-Colorectal (FACT-C) quality of life instrument. *Qual Life Res* **8**, 181-195 (1999).
6 Alrubaiy, L. *et al.* Development of a short questionnaire to assess the quality of life in Crohn's disease and ulcerative colitis. *J Crohns Colitis* **9**, 66-76, doi:http://dx.doi.org/10.1093/ecco-jcc/jju005 (2015).
7 Alcala, M. J., Casellas, F., Fontanet, G., Prieto, L. & Malagelada, J. R. Shortened questionnaire on quality of life for inflammatory bowel disease. *Inflamm Bowel Dis* **10**, 383-391 (2004).
8 Allen, P. B. *et al.* Development and validation of a patient-reported disability measurement tool for patients with inflammatory bowel disease. *Aliment Pharmacol Ther* **37**, 438-444, doi:http://dx.doi.org/10.1111/apt.12187 (2013).
9 Cheung, W. Y., Garratt, A. M., Russell, I. T. & Williams, J. G. The UK IBDQ-a British version of the inflammatory bowel disease questionnaire. development and validation. *Journal of Clinical Epidemiology* **53**, 297-306 (2000).
10 Drossman, D. A. *et al.* The rating form of IBD patient concerns: a new measure of health status. *Psychosom Med* **53**, 701-712, doi:10.1097/00006842-199111000-00010 (1991).
11 Guyatt, G. *et al.* A new measure of health status for clinical trials in inflammatory bowel disease. *Gastroenterology* **96**, 804-810 (1989).
12 Irvine, E. J., Zhou, Q. & Thompson, A. K. The Short Inflammatory Bowel Disease Questionnaire: a quality of life instrument for community physicians managing inflammatory bowel disease. CCRPT Investigators. Canadian Crohn's Relapse Prevention Trial. *Am J Gastroenterol* **91**, 1571-1578 (1996).
13 Peyrin-Biroulet, L. *et al.* Development of the first disability index for inflammatory bowel disease based on the international classification of functioning, disability and health. *Gut* **61**, 241-247, doi:10.1136/gutjnl-2011-300049 (2012).
14 Smith, J. J. *et al.* Development of a social morbidity score in patients with chronic ulcerative colitis as a potential guide to treatment. *Colorectal Dis* **14**, e250-257, doi:http://dx.doi.org/10.1111/j.1463-1318.2012.02880.x (2012).
15 Wilcox, A. R., Dragnev, M. C. C., Darcey, C. J. & Siegel, C. A. A new tool to measure the burden of Crohn's disease and its treatment: do patient and physician perceptions match? *Inflamm Bowel Dis* **16**, 645-650, doi:http://dx.doi.org/10.1002/ibd.21094 (2010).
16 Blazeby, J. M. *et al.* Development of a questionnaire module to supplement the EORTC QLQ-C30 to assess quality of life in patients with hepatocellular carcinoma, the EORTC QLQ-HCC18. *European Journal of Cancer* **40**, 2439-2444 (2004).
17 Heffernan, N. *et al.* Measuring health-related quality of life in patients with hepatobiliary cancers: the functional assessment of cancer therapy-hepatobiliary questionnaire. *J Clin Oncol* **20**, 2229-2239 (2002).
18 Yount, S. *et al.* Assessment of patient-reported clinical outcome in pancreatic and other hepatobiliary cancers: the FACT Hepatobiliary Symptom Index. *J Pain Symptom Manage* **24**, 32-44 (2002).
19 Kavadas, V. *et al.* Development of an EORTC disease-specific quality of life questionnaire for use in patients with liver metastases from colorectal cancer. *European Journal of Cancer* **39**, 1259-1263 (2003).

20 Blazeby, J. M. *et al.* Validation of the European Organization for Research and Treatment of Cancer QLQ-LMC21 questionnaire for assessment of patient-reported outcomes during treatment of colorectal liver metastases. *Br J Surg* **96**, 291-298, doi:http://dx.doi.org/10.1002/bjs.6471 (2009).
21 Anderson, R. T. *et al.* Development and initial psychometric evaluation of the hepatitis C virus-patient-reported outcomes (HCV-PRO) instrument. [References]. *Quality of Life Research: An International Journal of Quality of Life Aspects of Treatment, Care & Rehabilitation* **23**, 561-570, doi:http://dx.doi.org/10.1007/s11136-013-0505-7 (2014).
22 Gralnek, I. M. *et al.* Development and evaluation of the Liver Disease Quality of Life instrument in persons with advanced, chronic liver disease--the LDQOL 1.0. *Am J Gastroenterol* **95**, 3552-3565 (2000).
23 Younossi, Z. M., Guyatt, G., Kiwi, M., Boparai, N. & King, D. Development of a disease specific questionnaire to measure health related quality of life in patients with chronic liver disease. *Gut* **45**, 295-300 (1999).
24 van der Plas, S. M. *et al.* The Liver Disease Symptom Index 2.0; Validation of a disease-specific questionnaire. *Quality of Life Research: An International Journal of Quality of Life Aspects of Treatment, Care & Rehabilitation* **13**, 1469-1481, doi:http://dx.doi.org/10.1023/B:QURE.0000040797.17449.c0 (2004).
25 Wassef, W., Bova, C., Barton, B. & Hartigan, C. Pancreatitis Quality of Life Instrument: Development of a new instrument. *SAGE Open Med* **2**, 2050312114520856, doi:http://dx.doi.org/10.1177/2050312114520856 (2014).
26 Wassef, W. *et al.* Pancreatitis Quality of Life Instrument: A Psychometric Evaluation. *Am J Gastroenterol* **111**, 1177-1186, doi:http://dx.doi.org/10.1038/ajg.2016.225 (2016).
